# Potential Therapeutic Effect of Traditional Chinese Medicine on Coronavirus Disease 2019: A Review

**DOI:** 10.3389/fphar.2020.570893

**Published:** 2020-11-09

**Authors:** Qin Qiu, Yuge Huang, Xiaohua Liu, Fangfang Huang, Xiaoling Li, Liao Cui, Hui Luo, Lianxiang Luo

**Affiliations:** ^1^ Graduate School, Guangdong Medical University, Zhanjiang, China; ^2^ Department of Pediatrics, The Affiliated Hospital of Guangdong Medical University, Zhanjiang, China; ^3^ Animal Experiment Center, Guangdong Medical University, Zhanjiang, China; ^4^ Guangdong Key Laboratory for Research and Development of Natural Drugs, Guangdong Medical University, Zhanjiang, China; ^5^ The Marine Biomedical Research Institute, Guangdong Medical University, Zhanjiang, China; ^6^ The Marine Biomedical Research Institute of Guangdong Zhanjiang, Zhanjiang, China

**Keywords:** SARS-CoV-2, COVID-19, traditional Chinese medicine, therapeutic effect, technology

## Abstract

The Coronavirus disease 2019 (COVID-19) pandemic caused by severe acute respiratory syndrome coronavirus 2 has been rapidly spreading globally and has caused worldwide social and economic disruption. Currently, no specific antiviral drugs or clinically effective vaccines are available to prevent and treat COVID-19. Traditional Chinese medicine (TCM) can facilitate syndrome differentiation and treatment according to the clinical manifestations of patients and has demonstrated effectiveness in epidemic prevention and control. In China, TCM intervention has helped to control the epidemic; however, TCM has not been fully recognized worldwide. In this review, we summarize the epidemiology and etiological characteristics of severe acute respiratory syndrome coronavirus 2 and the prevention and treatment measures of COVID-19. Additionally, we describe the application of TCM in the treatment of COVID-19 and the identification of small molecules of TCM that demonstrate anti-coronavirus activity. We also analyze the current problems associated with the recognition of TCM. We hope that, through the contribution of TCM, combined with modern technological research and the support of our international counterparts, COVID-19 can be effectively controlled and treated.

Coronavirus disease 2019 (COVID-19) caused by severe acute respiratory syndrome coronavirus 2 (SARS-CoV-2), a novel and pathogenic coronavirus, has developed into a public health emergency of international concern (World Health Organization, 2020). As of August 6, 2020, more than 19 million confirmed cases have been reported across more than 216 countries and territories, resulting in more than 700,000 deaths (according to data from Johns Hopkins University) and causing a great negative impact on people’s health and economic development. COVID-19 is the worst global health crisis since the Spanish flu pandemic of 1918, and no specific antiviral agent or effective vaccine has been found (Gupta et al., 2020). As the global COVID-19 pandemic continues to escalate rapidly, an urgent need exists to identify safe and effective drugs or potential adjuvant therapy. Accordingly, we briefly review the epidemiology, pathogenesis and key targets, multi-organ damage and conventional preventive treatment of SARS-CoV-2, focusing on the application of traditional Chinese medicine (TCM) in the treatment of COVID-19 patients. Additionally, some opinions on the difficulties and solutions to the modernization of TCM in China are expressed.

## Epidemiology of Severe Acute Respiratory Syndrome Coronavirus 2

Understanding the epidemiology of this virus is a key element to develop strategies for preventing COVID-19. Based on the findings of phylogenetic analysis, SARS-CoV-2 may have originated from bats or bat droppings associated with pollutants in the market or surrounding areas (Wu F. et al., 2020). SARS-CoV-2 has three types of hosts (natural, intermediate, and final) that can be transmitted between human hosts via respiratory droplets and contact routes (Li Q. et al., 2020). Moreover, the existing evidence of SARS-CoV-2 infecting intestinal epithelial cells reminds us to focus on the possibility of fecal-oral transmission (Lamers et al., 2020). Both asymptomatic and symptomatic patients are communicators; however, in the case of symptomatic patients, an increased viral load was observed (Kim et al., 2020). Studies have shown that adults are more susceptible to infection than children, especially the elderly with basic diseases such as hypertension and diabetes, among whom 80.9% have mild to moderate disease, and the mortality rate of confirmed cases is about 2.3% (Chang et al., 2020; Wu and McGoogan, 2020).

## Pathogenesis and Key Targets of Severe Acute Respiratory Syndrome Coronavirus 2

**FIGURE 1:**
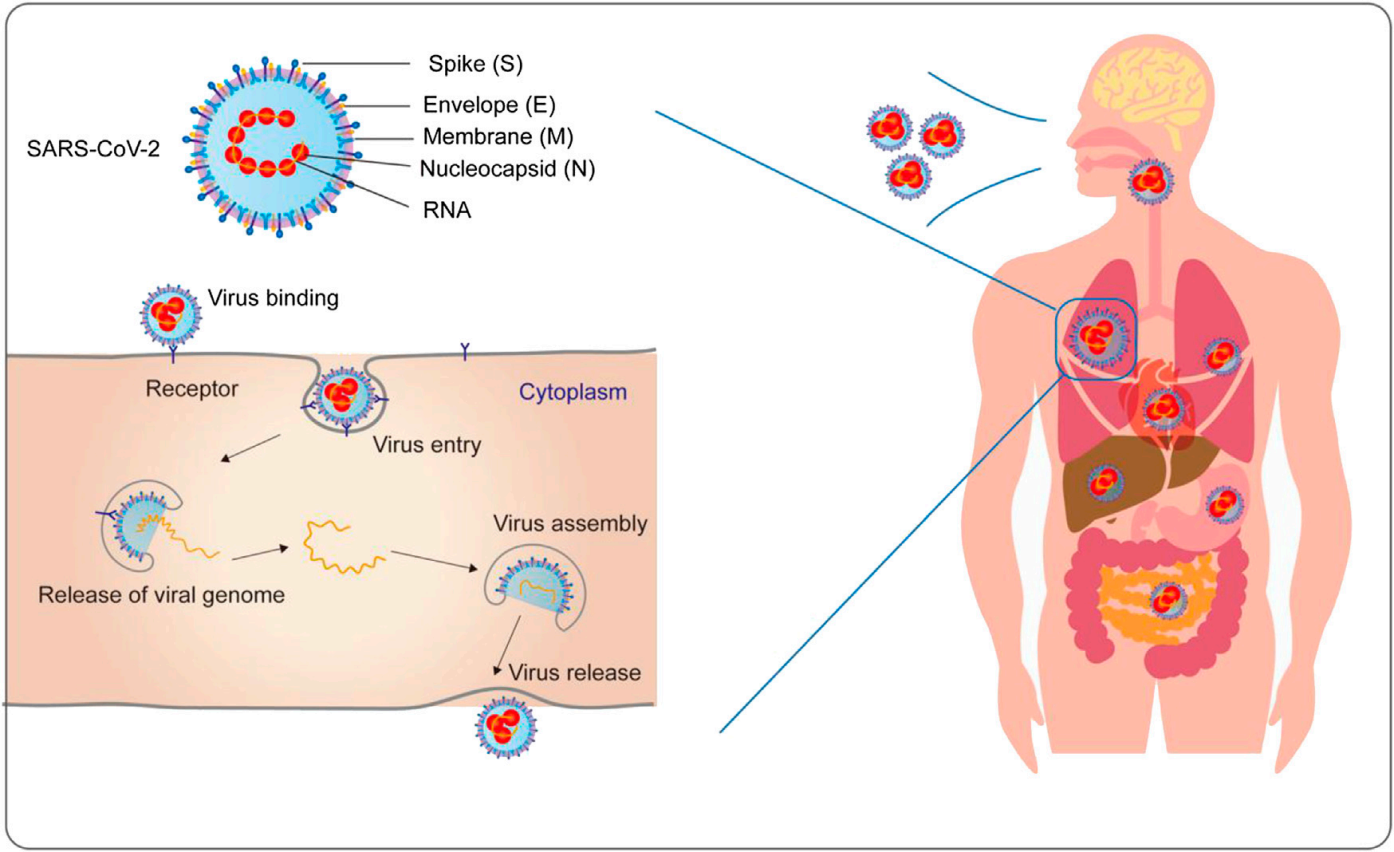
Severe Acute Respiratory Syndrome Coronavirus 2 (SARS-COV-2) is an enveloped RNA virus, comprising RNA and four major structural proteins [the nucleocapsid (N) protein, membrane (M) glycoprotein, envelope (E) protein, and spike (S) glycoprotein]. The pathogenic mechanism includes the four steps: attachment and entry, replication and transcription, assembly, and release. The virus mainly invades the host through the respiratory tract and directly or indirectly causes systemic multiple organ damage by identifying specific receptors on the host cell membrane.

SARS-CoV-2 is a positive-sense, single-stranded RNA virus with a genome of 29.9 kb and a diameter ranging from 80 to 160 nm, and is a novel beta-coronavirus belonging to the *Sarbecovirus* subgenus of the *Coronaviridae* family(Chan et al., 2020; Zhu N. et al., 2020). Its structure comprises a helical nucleocapsid formed by the binding of nucleic acid to the nucleocapsid (N) protein and a lipid envelope studded with structural proteins, including the membrane (M) glycoprotein, envelope (E) protein, and spike (S) glycoprotein (Boopathi et al., 2020). The pathogenic mechanism of coronavirus includes four steps: attachment and entry, replication and transcription, assembly and release (Fehr and Perlman, 2015). SARS-CoV-2 binds to the receptor angiotensin converting enzyme 2 (ACE2) with the help of S protein to enter cells and releases RNA that translates two polyproteins and structural proteins; thereafter, the viral genome begins to replicate (Yan R. et al., 2020). Genomic RNA and nucleocapsid proteins combine to form nucleocapsids, and then the vesicles containing the virus particles fuse with the plasma membrane to release the virus (Figure 1) (Knoops et al., 2008; Fehr and Perlman, 2015). SARS-CoV-2 S proteins recognize ACE2 for entry and the serine protease TMPRSS2 for S protein priming (Hoffmann et al., 2020). Nevertheless, more studies have focused on nonstructural proteins such as the papain-like protease (PLpro), the 3C-like protease (3CLpro) and the RNA-dependent RNA polymerase (RdRp), which are critical for viral replication (Dong et al., 2020). These key proteins could be potential targets for diagnostic or therapeutic application.

## Multi-Organ Damage of Severe Acute Respiratory Syndrome Coronavirus 2

**FIGURE 2:**
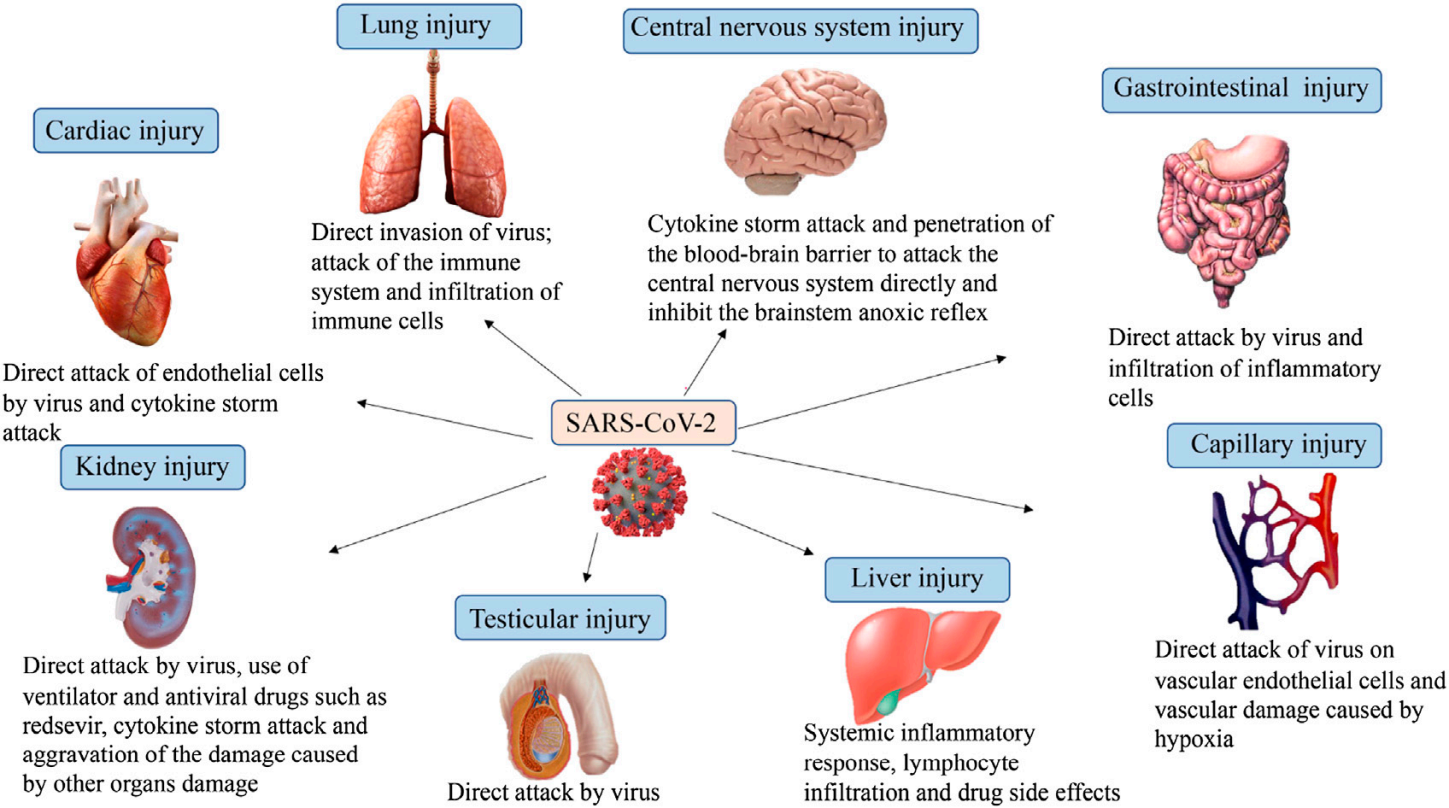
Multi-organ dysfunction caused by SARS-CoV-2 infection includes heart injury, kidney injury, liver injury, lung injury, central nervous system injury, testicular injury, capillary injury, and gastrointestinal injury. Most organ damage is caused by direct virus attack and cytokine storm attack.

After infecting the host, the SARS-CoV-2 stimulates humoral and cellular immunity, causing cytokine storms, which trigger a violent attack by the body’s immune system (Sun et al., 2020). ACE2 has been identified as the functional host receptor for SARS-CoV-2 and is widely expressed in various human organs, including the oral and nasal mucosa, nasopharynx, lung, stomach, small intestine, skin, spleen, liver, kidney, and brain (Figure 2) (Li M.-Y. et al., 2020; Wang Q. et al., 2020). Among them, ACE2 immunostaining is the most abundant in alveolar epithelial cells; thus, the lung is a target organ that is most easily affected (Hamming et al., 2004). Autopsy reports from China and the United States both revealed diffuse alveolar injury and chronic inflammatory edema of the bronchial mucosa, confirming the occurrence of ARDS in COVID-19 patients whose main clinical manifestations are fever, cough and progressive dyspnea (Barton et al., 2020; Tian et al., 2020). Histological analysis of pulmonary vessels in patients with COVID-19 showed extensive thrombosis with microvascular lesions, with the amount of new vessel growth 2.7 times higher than that in patients with influenza (Ackermann et al., 2020). ACE2 is highly expressed not only in lung cells but also throughout the gastrointestinal tract (Bourgonje et al., 2020). Some patients experience gastrointestinal symptoms, including vomiting, abdominal pain and diarrhea (Cheung et al., 2020; Cholankeril et al., 2020), abdominal pain and diarrhea; imaging findings have suggested intestinal abnormalities (Bhayana et al., 2020). A case report revealed that SARS-CoV-2 enterocolitis continues to expel the virus for approximately two weeks after recovering from diarrhea (Hosoda et al., 2020), while studies from China have also found viral nucleocapsid protein in gastric, duodenal, and rectum glandular epithelial cells (Xiao et al., 2020). However, more evidence is needed to determine whether the virus has the possibility of fecal-oral transmission (Wu Y. et al., 2020). Several reports have indicated that the elevation of transaminase in patients suggests liver damage should be given attention (Feng et al., 2020; Wang Y. et al., 2020). SARS-CoV-2 infection is also associated with various diseases of the cardiovascular system, including myocarditis, cardiomyopathy and excessive vasoconstriction (Craver et al., 2020; Moderato et al., 2020). Some critically ill patients have abnormal hemagglutination with high D-dimer levels and elevated fibrinogen, which may cause vascular embolism, resulting in pulmonary embolism and stroke (Hess et al., 2020; Lax et al., 2020). If the virus appears in cutaneous blood vessels in patients with COVID-19, they may cause corresponding pathologic changes (Gianotti et al., 2020; Llamas-Velasco et al., 2020; Torres and Puig, 2020). The presence of the virus in the nerve and capillary endothelial cells in the frontal lobe tissue of an infected patient indicates that the virus can penetrate the blood-brain barrier to attack the central nervous system directly (Paniz-Mondolfi et al., 2020; Reichard et al., 2020). Therefore, some patients have nervous system manifestations such as anosmia, dysgeusia, ataxia, and an altered mental status (Baig, 2020). Additionally, research data have shown that the mortality of patients with COVID-19 is related to the prevalence of kidney disease on admission (Cheng et al., 2020), and virus particles have been found in renal tubular epithelial cells under ultrastructure, providing evidence of direct infection of the kidney by SARS-CoV-2 (Farkash et al., 2020). In addition to the direct virulence of SARS-CoV-2, factors contributing to acute kidney injury include systemic hypoxia, abnormal coagulation, and possible drug or hyperventilation-relevant rhabdomyolysis (Su et al., 2020). Notably, Xixi Liu et al. found that ACE2-expressing cells exist in almost all testicular cell types, and sertoli cells have the highest expression level and positive cell ratio (Liu X. et al., 2020). Zhengpin Wang et al. also confirmed that the human testis is a potential target for SARS-CoV-2 from the level of single-cell transcription (Wang and Xu, 2020). Thus, SARS-CoV-2 causes damage to multiple organs of the host through various factors, especially hypertension, the elderly, obesity, male individuals with severe cardiovascular disease and those with blood group A (Menter et al., 2020). Therefore, the integration of multiple disciplines to carry out comprehensive diagnoses and treatment for patients with COVID-19 is warranted.

## Conventional Preventive Treatment of Severe Acute Respiratory Syndrome Coronavirus 2

Although SARS-CoV-2 is widespread and causes multiple organ damage, no specific antiviral drugs or vaccines are currently available. Prevention and supportive care are the main treatment strategies for COVID-19 (Jin et al., 2020). As one of the first affected countries, the epidemic in China has been well controlled, proving the importance of non-drug intervention (NPI), including the isolation of ill persons, contact tracing, quarantine of exposed persons, travel restrictions, school and workplace closures, cancellation of mass gatherings, and hand washing, among others (Lai et al., 2020). At present, there are mainly the following categories of drugs used to treat COVID-19, including antiviral drugs (e.g., remdesivir), antibodies (e.g., convalescent plasma), anti-inflammatory drugs (e.g., dexamethasone), and targeted immunomodulatory therapy (e.g., tocilizumab) (Gautret et al., 2020). Most antiviral drugs currently used to treat COVID-19 were initially developed for influenza, Ebola, or SARS/MERS. Convalescent plasma might be a potential therapy for critically ill patients infected with SARS-CoV-2 (Zhang and Liu, 2020). As a monoclonal antibody against inflammatory cytokines, tocilizumab has emerged as an alternative treatment for COVID-19 patients with a risk of cytokine storms (Luo P. et al., 2020). In addition, for the use of corticosteroids in the treatment of COVID-19, a study has been found that corticosteroids can reduce pulmonary immune inflammatory responses, but delay viral clearance, and systemic use is not recommended in patients with SARS-CoV-2 infection (Russell et al., 2020). However, another controlled, open-label trial found that dexamethasone use resulted in lower 28-days mortality in patients randomized to invasive mechanical ventilation or oxygen alone, compared with no benefit in patients with shorter duration of symptoms and no need for supplemental oxygen (Horby et al., 2020). The development of vaccines is the key to halting the spread of the virus. Thus, clinical trials have been carried out in many countries, among which the fastest progress concerns adenovirus vector vaccines, mRNA vaccines, DNA vaccines and inactivated vaccines, which have entered the clinical stage (Amanat and Krammer, 2020).

## Application of Traditional Chinese Medicine to Prevent and Treat Coronavirus Disease 2019

According to the History of Chinese Epidemics published by the Chinese Academy of Traditional Chinese Medicine, 321 epidemics have occurred in China in the past 2000 years since the Western Han Dynasty and have been resisted effectively by TCM (Medicine, 2003.06). The efficacy of TCM has been confirmed in the process of fighting severe acute respiratory syndrome (SARS) in 2003 (Chen and Nakamura, 2004; Zhang et al., 2004). TCM has achieved phased victories in China. According to statistics released by the China Administration of Traditional Chinese Medicine, 92.58% of the confirmed COVID-19 patients nationwide were treated with TCM, and the effective rate of TCM participation reached 95.10% up to March 3 (Medicine NAoTC, 2020h). TCM has therapeutic effects on all clinical stages of COVID-19 (Xiang et al., 2020). For mild and ordinary stages, TCM can relieve clinical symptoms (fever, shortness of breath, cough, diarrhea), shorten the course of the disease, improve the cure rate, and prevent the disease from becoming severe. For severe and critical cases, combined with supportive therapy such as oxygen therapy and fluid replacement in modern medicine, TCM can enhance pulmonary ventilation function and inhibit lung injury and an excessive immune response. In the rehabilitation stage, patients usually still have deficiencies in qi and yin. Thus, taking TCM compound decoctions with the effects of supplementing qi, nourishing yin and strengthening the spleen and tonifying the lung can help to restore physical strength, promote the absorption of pulmonary inflammation and reduce pulmonary interstitial fibrosis (Ang et al., 2020a; Liu, 2020). The successful experience of fighting COVID-19 has shown that TCM has a unique advantage in infectious diseases.

### IDENTIFICATION OF ANTI-NOVEL CORONAVIRUS COMPOUNDS ISOLATED FROM TRADITIONAL CHINESE MEDICINE

The development of new agents against COVID-19 is not realistic to pass safety and toxicity tests in a short period and is both time-consuming and costly. Therefore, active compounds targeting viruses or host targets in existing Chinese herbal medicines were screened by many scientists. Currently, various natural compounds have been found to potentially possess anti-SARS-CoV-2 activity. In this review, we focus on six compounds: hesperetin, baicalin, curcumin, glycyrrhizic acid, luteolin, and rutin. The pharmacological action and targeting targets of these six compounds are shown in Table 1.

**TABLE 1:** Compounds with anti-coronavirus activity.

Compounds	Pharmacological action	Mode of action	References
Hesperetin	Antioxidation, anti-inflammation	3CLpro, Mpro, RBD- Spro	(Parhiz et al., 2015; Adem et al., 2020)
Baicalin	Anti-inflammatory, antioxidant, neuro-protective	ACE2	(Hwang et al., 2005; Chen and Du, 2020b)
Curcumin	Antioxidant, anti-inflammatory, anti-virus, anti-cancer	PLpro, RBD-Spro, protease domain-ACE2	(Moghadamtousi et al., 2014; Goswami et al., 2020)
Glycyrrhizic acid	Antiviral, antioxidant, immunomodulatory, cell membrane stabilization	ACE2	(Pang et al., 2016; Chen and Du, 2020b)
Luteolin	Antioxidation, anti-inflammation, anti-tumor	3CLpro, RdRp, PLpro, Spro	(Xiong et al., 2017; Yu et al., 2020)
Rutin	Anti-inflammation, anti-virus, anti-oxidation, neuroprotective effects	Mpro	(Chua, 2013; Huynh et al., 2020)

#### Hesperetin

Hesperetin, a flavonoid from the pericarp of citrus, has the biological characteristics of antioxidation and anti-inflammation (Parhiz et al., 2015). Hesperetin inhibited the cleavage activity of SARS-CoV 3C-like protease (3CLpro) in a dose-dependent manner, in which the IC_50_ was 8.3 μmol/l in the Vero cell lysis assay (Lin et al., 2005). Using computer virtual screening, hesperetin showed good binding affinity for 3CLpro (Wu et al., 2020a). Additionally, it binds well to COVID-19 Main protease (Mpro) (Adem et al., 2020) and may destroy the interaction between the receptor-binding domain (RBD) and ACE2 by binding to RBD in SARS-CoV-2 S protein (Wu et al., 2020a).

#### Baicalin

Baicalin is a flavonoid compound extracted and isolated from *Scutellaria baicalensis* Georgi, which has multiple biological activities, including anti-inflammatory, antioxidant, and neuro-protective effects (Hwang et al., 2005; Shi et al., 2017; Guo et al., 2019). Scientists have proven that baicalin has anti-SARS-CoV virus activity *in vitro* using the fetal rhesus kidney-4 (fRhK-4) cell line, in which the EC_50_ was 12.5 μg/ml at 48 h and the Vero-E6 cell line in which the EC_50_ was 100 μg/ml at 48 h; in the plaque reduction assay, using Vero cell line, the EC_50_ of baicalin was 11 μg/ml (Chen et al., 2004). The results of molecular docking showed that baicalin has a strong binding affinity to the ACE2 receptor (Chen and Du, 2020b).

#### Curcumin

Curcumin, a known phenolic compound extracted from the rhizome of turmeric, showed broad pharmacological effects, including antioxidant, anti-inflammatory, anti-virus and anti-cancer activities (Anand et al., 2008; Aggarwal and Sung, 2009; Moghadamtousi et al., 2014). Curcumin has activity against SARS-CoV replication with concentrations between 3.3 and 10 μmol/l, and inhibitory effects on 3CLpro were observed with IC_50_ values of 40 μmol/l (Wen et al., 2007). The release of IL-1, IL-6 and TNF-α in the treatment of patients with a cytokine storm was blocked by curcumin (Sordillo and Helson, 2015). The results of molecular docking using virtual screening showed that curcumin possess good affinity to PLpro, the RBD of spike glycoprotein (RBD-S), and the ACE2 receptor at the protease domain (PD-ACE2) (Goswami et al., 2020; Utomo and Meiyanto, 2020).

#### Glycyrrhizic Acid

Glycyrrhizic, also called glycyrrhizin, is one of the most important active ingredients in *Glycyrrhiza glabra* L., which has many biological activities such as antiviral, antioxidant, immunomodulatory and cell membrane stabilization (Yong and Haji, 2010; Akman et al., 2015; Pang et al., 2016). In addition to inhibition of SARS-CoV replication, glycyrrhizin inhibits the adsorption and penetration of the virus and is less effective during the adsorption period (EC_50_, 600 mg/l) and after the virus adsorption period (EC_50_, 2,400 mg/l) and most effective both during and after the adsorption period (Cinatl et al., 2003). Chen et al. used systems biology tools to identify that a novel combination of vitamin C, curcumin and glycyrrhizic acid (VCG Plus) may regulate the immune response against SARS-CoV-2 infections by acting on NOD-like and Toll-like signaling pathways and inhibit excessive inflammatory responses to prevent the onset of a cytokine storm by inhibiting PI3K/AKT, NF-κB and MAPK signaling pathways (Chen L. et al., 2020). The activity of ACE2 in cells may be regulated by the binding of glycyrrhizin to ACE2, 3CLpro, Spike, PLpro, and RdRp (Huang F. et al., 2020; Chen and Du, 2020b).

#### Luteolin

As a natural flavonoid, luteolin not only has multiple effects such as antioxidation and anti-inflammation but also inhibits the proliferation of tumor cells (Xiong et al., 2017; Yu et al., 2019). Luteolin dose-dependently inhibited the cleavage activity of SARS coronavirus, and the EC_50_ value was 10.6 μmol/l (Yi et al., 2004). An *in vitro* study showed that luteolin could inhibit SARS-CoV 3CLPro, with an IC_50_ of 20.2 μmol/l (Ryu et al., 2010). Through molecular docking, luteolin showed strong interactions with the targets of SARS-CoV-2, including 3CLpro, RdRp, PLpro, and Spro (Yu et al., 2020).

#### Rutin

Rutin, a type of flavonoid, is an effective component of Lianhua Qingwen, which has many effects, such as anti-inflammation, anti-virus, anti-oxidation, and neuroprotective effects (Javed et al., 2012; Chua, 2013). Various RNA viruses, including influenza A virus (IAV) and enterovirus A71 (EV-A71), are inhibited by rutin (Savov et al., 2006; Lin et al., 2012). Molecular dynamics simulation suggests that rutin can bind stably to the Mpro’ pocket of SARS-CoV-2 and block the binding of substrate in space (Huynh et al., 2020).

Through the enumeration of several compounds, it was found that flavonoids (e.g., hesperetin, baicalin and luteolin) have shown outstanding in fighting against the COVID-19. Flavonoids, a class of secondary metabolites produced by plants in the process of long-term natural selection, have a variety of pharmacological activities including antiviral, anti-inflammatory, cardiovascular and cerebrovascular disease prevention, antioxidation and anti-tumor, etc (Panche et al., 2016; Brodowska, 2017). Its unique and common chemical structure, i.e., C6-C3-C6 consisting of 2 aromatic rings (A and B) linked by a three-carbon chain (Kumar and Pandey, 2013). The hydroxylation pattern of the B ring of certain flavonoids enhanced the inhibitory effect of mast cells and macrophages on cytokine secretion (Ginwala et al., 2019). Flavonoids exerts express their anti-inflammatory activity by inhibiting the synthesis and activity of pro-inflammatory mediators (e.g., eicosanoids and cytokines), inhibiting the activation of transcription factors (e.g., NF-kappaB and activating protein-1) and modulation of proinflammatory gene expression (Kim et al., 2004; Serafini et al., 2010). High affinity binding between flavonoids and S protein, helicase, and protease sites on ACE2 resulting in conformational change, thereby inhibiting viral entry of SRAS-CoV-2. In addition, saikosaponins have great potential in the treatment of COVID-19 through immunomodulatory, anti-inflammatory and antiviral activities. On the one hand, saikossaponins exhibit anti-inflammatory effect by dose-dependently inhibiting the production of several inflammatory mediators which are responsible for the cytokine storm of severe COVID-19 patients, and immunomodulatory effect by inhibiting the proliferation of activated T lymphocytes (Bahbah et al., 2020). On the other hand, it can also directly bind to ACE2 to play the role of anti-SARS-CoV-2 (Yan Y.-M. et al., 2020). In short, most of the naturally active compounds reviewed by us show antiviral and anti-inflammatory effects and may possess anti-SARS-CoV-2 effects according to the results of computer simulation and *in vitro* experiments, but further *in vivo* and clinical trials are needed to verify.

### APPLICATION OF ANTI- CORONAVIRUS DISEASE 2019 TRADITIONAL CHINESE MEDICINE HERBAL FORMULAS

In addition to compounds, many TCM herbal formulas are also widely used in the treatment of COVID-19. The formula therapy of TCM diagnosis and treatment has the advantages of multi-target and multi-link treatment. TCM herbal formulas such as Qingfei Paidu decoction, Huashi Baidu recipe, Lianhua Qingwen capsule and Xuebijing injection have demonstrated curative effects on COVID-19 (Table 2) (Medicine NAoTC, 2020h). Among them, the clinical stages are mainly based on the Guideline on Diagnosis and Treatment of Coronavirus disease 2019 in China (Medicine NAoTC, 2020f).

**TABLE 2:** Clinical application of traditional Chinese medicine in the treatment of Coronavirus disease 2019.

TCM herbal formulas	Constituent	Clinical stage	Therapeutic effect	References
Qing Fei pai Du decoction	*Ephedra sinica* Stapf, *Glycyrrhiza glabra* L., *Prunus amygdalus* Batsch, Gypsum Fibrosum, *Cinnamomum cassia* (L.) J.Presl, *Alisma plantago-aquatica subsp. orientale* (Sam.) Sam., *Polyporus umbellatus* (Pers) Fr., *Atractylodes macrocephala* Koidz., *Thespesia populnea* (L.) Sol. ex Corrêa, *Bupleurum falcatum* L., *Scutellaria baicalensis* Georgi, *Zingiber officinale* Roscoe, *Aster tataricus* L.f., *Tussilago farfara* L., *Iris domestica* (L.) Goldblatt and Mabb., *Asarum sieboldii* Miq., *Dioscorea alata* L., *Citrus × aurantium* L., *Pogostemon cablin* (Blanco) Benth	mild, ordinar, severe, critical	“Clear the lung and calm panting” according to TCM theory. Reportedly has anti-inflammatory and lung injury reduction effects	(Xu et al., 2020)
Hua Shi Bai Du recipe	*Ephedra sinica* Stapf, *Pogostemon cablin* (Blanco) Benth., Gypsum Fibrosum, *Prunus amygdalus* Batsch, *Pinellia ternata* (Thunb.) Makino, *Magnolia officinalis* Rehder and E.H.Wilson, *Atractylodes lancea* (Thunb.) DC., *Lanxangia tsao-ko* (Crevost and Lemarié) M.F.Newman and Skornick., *Thespesia populnea* (L.) Sol. ex Corrêa, *Astragalus mongholicus* Bunge, *Paeonia lactiflora* Pall., *Descurainia sophia* (L.) Webb ex Prantl, *Rheum officinale* Baill., *Glycyrrhiza glabra* L	mild, ordinary, severe	“Clear heat and detoxifying, removing dampness” according to TCM theory. Reportedly has cough symptom relief effect	(Chinadaily, 2020; Medicine NAoTC, 2020b)
Huo Xiang Zheng Qi powder	*Perilla frutescens* (L.) Britton, *Thespesia populnea* (L.) Sol. ex Corrêa, *Pinellia ternata* (Thunb.) Makino, *Atractylodes macrocephala* Koidz., *Citrus × aurantium* L., *Areca catechu* L., *Angelica dahurica* (Hoffm.) Benth. and Hook.f. ex Franch. and Sav., *Magnolia officinalis* Rehder and E.H.Wilson, *Platycodon grandiflorus* (Jacq.) A.DC., *Pogostemon cablin* (Blanco) Benth., *Glycyrrhiza glabra* L	mild, ordinary	“Harmonize the exterior and interior, and remove dampness” according to TCM theory. Reportedly has anti-inflammation, immune protection and gastrointestinal motility regulation effects	(Zhao et al., 2019)
Jin Hua Qing Gan granule	*Lonicera japonica* Thunb., Gypsum Fibrosum, *Ephedra sinica* Stapf, *Prunus amygdalus* Batsch, *Scutellaria baicalensis* Georgi, *Forsythia suspensa* (Thunb.) Vahl, *Fritillaria thunbergii* Miq., *Anemarrhena asphodeloides* Bunge, *Arctium lappa* L., *artemisia annua* L., *Mentha × piperita* L., *Glycyrrhiza glabra* L	mild, ordinary	“Clear heat and detoxifying, and diffuse the lung” according to TCM theory. Reportedly has antiviral and immune regulation effects	(Jimilihan, 2020)
Lian Hua Qing Wen capsule	*Forsythia suspensa* (Thunb.) Vahl, *Lonicera japonica* Thunb., *Ephedra sinica* Stapf, *Isatis tinctoria* L., Gypsum Fibrosum, *Mentha × piperita* L., *Pogostemon cablin* (Blanco) Benth., *Houttuynia cordata* Thunb., *Rheum officinale* Baill., *Prunus amygdalus* Batsch, *Glycyrrhiza glabra* L	mild, ordinary	“Clear heat and diffuse the lung, and detoxifying” according to TCM theory. Reportedly has antiviral, anti-inflammatory and immune regulation effects	(Ye et al., 2020)
Xuan Fei Bai Du granule	*Ephedra sinica* Stapf, *Prunus amygdalus* Batsch, *Coix lacryma-jobi* L., *Atractylodes macrocephala* Koidz., *Pogostemon cablin* (Blanco) Benth., *artemisia annua* L., Gypsum Fibrosum, *Reynoutria japonica* Houtt., *Verbena officinalis* L., *Phragmites australis subsp. australis*, *Citrus maxima* (Burm.) Merr., *Descurainia sophia* (L.) Webb ex Prantl, *Glycyrrhiza uralensis* Fisch. ex DC.	mild, ordinary	“Detoxify and remove blood stasis, diffuse the lung, removing dampness, clear heat” according to TCM theory	(Chinadaily, 2020)
Xue Bi Jing injection	*Carthamus tinctorius* L., *Paeonia lactiflora* Pall., *Conioselinum anthriscoides “Chuanxiong”*, *Salvia miltiorrhiza* Bunge, *Angelica sinensis* (Oliv.) Diels	Severe, critical	“Dissolve stasis and detoxifying.” Immune regulation	(Chen et al., 2018)
Shen Fu injection	*Panax ginseng* C.A.Mey., *Aconitum carmichaeli* Debeaux	Severe, critical	“Restoring yang to rescue collapse and replenishing qi to prevent collapse.” Reportedly has anti-inflammatory and immune regulation effects	(Liu et al., 2019)
Tan Re Qing injection	*Scutellaria baicalensis* Georgi, Fel Ursi, *Lonicera japonica* Thunb., Corne Caprae Hirci, *Forsythia suspensa* (Thunb.) Vahl	Severe, critical	“Clear heat and detoxifying, and resolving phlegm” according to TCM theory. Reportedly has antiviral, anti-inflammatory and immune regulation effects	(Jiang et al., 2009)

#### Jin Hua Qing Gan Granule

Jin Hua Qing Gan granule (JHG) have antiviral and immune regulation effects (Jimilihan, 2020). However, according to the current study, JHG plays an anti-COVID-19 effect mainly through modulating cytokine storm associated with COVID-19 mortality and acting directly on the virus itself (Ruan et al., 2020). The arachidonic acid (AA) metabolic pathway is mainly used to synthesize inflammatory cytokines; thus, inhibiting the AA metabolic pathway is beneficial to reduce the “cytokine storm.” Ren Y et al. showed that JHG might be anti-COVID-19 by treating the cytokine storm based on the AA metabolic pathway (Ren et al., 2020). A recent retrospective analysis test showed JHG can effectively shorten the duration of nucleic acid detection and promote the absorption of pneumonia inflammatory exudate without obvious adverse reactions in patients with COVID-19 (Liu Z. et al., 2020). A systematic analysis of multiple prospective randomized controlled trials provide evidence to determine that JHG is an efficacy and safety treatment for COVID-19 (Chen H. et al., 2020). JHQ is mainly used for COVID-19 patients with fever, cough, fatigue, headache and runny nose (Zhuang et al., 2020). At present, no adverse reactions reported. According to the recommended dosing methods of National Administration of Traditional Chinese Medicine, JHG is dissolved in boiling water and a bag or two 3 times a day for 5 or 7 days (Medicine NAoTC, 2020c).

#### Lian Hua Qing Wen Capsule

Lian Hua Qing Wen capsule (Lianhua Qingwen) is developed from the two classical formulas Fang Ma Xing Shi Gan Tang and Yinqiao Powder. In addition to improving the clinical symptoms of COVID-19 patients through anti-inflammation, Lianhua Qingwen can also act directly on the virus itself. *In vitro* studies have shown that Lianhua Qingwen can significantly inhibit the replication of SARS-CoV-2 in Vero E6 cells and significantly reduce the production of proinflammatory cytokines such as TNF-α, IL-6, CCL-2/MCP-1 and CXCL-10/IP-10. On the other hand, it affects the morphology of the virus at the mRNA level (Runfeng et al., 2020). The efficacy of Lianhua Qingwen in the treatment of H1N1 infection is similar to that of oseltamivir in terms of disease duration and virus shedding (Duan et al., 2011). A prospective, multicenter, open-label, randomized controlled trial including 284 confirmed COVID-19 (142 each in the treatment and control groups) displayed that add-on Lianhua Qingwen led to a shorter recovery time of fever, fatigue and coughing, a higher rate of improvement in chest computed tomographic manifestations and higher clinical cure; meanwhile, no serious adverse reactions were reported (Hu et al., 2020). Furthermore, Lianhua Qingwen was effective in improving clinical symptoms such as fever, shortness of breath, anorexia, fatigue and cough in COVID-19 patients, reported on two retrospective cohorts (Lu et al., 2020; Yao et al., 2020). In summary, Lianhua Qingwen was effective in improving the fever, cough, and fatigue of COVID-19, and no serious or adverse drug reactions were reported. The recommended dose is four capsules three times a day for 14 days (Hu et al., 2020; Medicine NAoTC, 2020c).

#### Xue Bi Jing Injection

The main components of Xue Bi Jing injection (XBJ) are *Carthamus tinctorius* L., *Paeonia lactiflora* Pall., *Conioselinum anthriscoides “Chuanxiong,” Salvia miltiorrhiza* Bunge and *Angelica sinensis* (Oliv.) Diels, which have been widely used for sepsis with no significant adverse events (Yin and Li, 2014). XBJ can not only anti-inflammatory but also direct action on SARS-CoV-2 in treating COVID-19. In terms of anti-inflammatory, XBJ injection reduces inflammatory reaction by down-regulating the expression of TLR4 and NF-κB, preventing lung injury caused by DDVP poisoning (He et al., 2018). XBJ improves survival in septic shock, and its mechanism is also related to inhibition of the immune response by preventing cytokine storm attack and regulating the balance of Tregs/Th17 cells (Chen et al., 2018). Therefore, treatment of COVID-19 with XBJ may be related to cytokine storm treatment (Ren et al., 2020). In terms of acting on the virus, *in vitro* experiments showed that XBJ could block the proliferation of SARS-CoV-2 and protect cells from SARS-CoV-2-induced cell death (Wen et al., 2020). Treating severe community-acquired pneumonia with XBJ can improve the pneumonia severity index, reduce the mortality, reduce the time of mechanical ventilation and shorten the hospitalization time of ICU stay (Song et al., 2019). Randomized controlled clinical trials revealed that XBJ may improve lung injury in patients with severe or critical COVID-19 (Wen et al., 2020). Another clinical research showed that, XBJ can effectively improve the inflammatory markers (such as white blood cell, lymphocyte count and C-reactive protein) and prognosis of severe COVID-19 patients (Wen et al., 2020). XBJ is mainly applied to treat sepsis, infection-induced systemic inflammatory response syndrome, and multiple organ dysfunction syndrome (Medicine NAoTC, 2020c). Now, there was no reports of any adverse reactions to the treatment. Its usage and dosage is 100 ml XBJ plus 100 ml 0.9% sodium chloride injection for intravenous drip every 12 h for 7 days(Ma et al., 2020b; Medicine NAoTC, 2020c).

#### Qing Fei Pai Du Decoction

Qing Fei Pai Du decoction (QFPDD) is made by adding and subtracting Ma Xing Shigan Tang, Wuling San, Shegan Mahuang Tang and Xiao Chaihu Tang in Zhang Zhongjing’s “typhoid fever.” Among them, Ma Xing Shigan decoction interferes with SARS-CoV-2 infection by regulating various complement and coagulation cascades and the thrombin system *in vivo* (Yang et al., 2020). The mechanism of QFPDD against COVID-19 may be related to the regulation of anti-viral, anti-inflammatory activity and metabolic programming. Recently, clinical observation in four provinces of China showed that the total effective rate of Qingfei Paidu decoction in the treatment of COVID-19 patients is more than 90%, in which more than 60% of the patients' symptoms and imaging manifestations improved significantly (n = 214) (Medicine NAoTC, 2020g). Observation of the curative effect of 1,262 cases of COVID-19 in 66 designated units in China demonstrated that it blocks disease progression in critical patients (Medicine NAoTC, 2020e). QFPDD decoction, a only general prescription, can treat all stages (light, ordinary, severe and critical), which has the characteristics of definite curative effect, convenient use, no side effects, and low cost (Medicine NAoTC, 2020d). Recommended treatment options is one dose daily with half of the dose taken in the morning and half in the evening (40 min after meal) with warm water for 3 days, and a course of three doses. The first course of treatment should use the original prescription. If the symptoms improve but are not cured, take the second course, in which the prescription can be modified according to the actual situation. The treatment should be stopped with the symptoms disappear (National Health Commission, 2020; Medicine NAoTC, 2020d).

#### Other Formulas

The theory in Traditional Chinese Medicine that “lung being connected with large intestine” is associated with the gut-lung axis. TCM alleviates and cures lung diseases by regulating the balance of the intestinal microenvironment of COVID-19 patients (Luo et al., 2020b). Huo Xiang Zheng Qi powder has therapeutic effects on gastrointestinal diseases (Zhao et al., 2018). The plaque reduction test explained that Liu Shen capsule (LS) could significantly inhibit the replication of SARS-CoV-2 in Vero E6 cells (IC_50_ = 0.6024 μg/ml) and reduce the production of pro-inflammatory cytokines, a finding that may be related to the regulation of the expression of key proteins in the NF-κB/MAPK signaling pathway (Ma et al., 2020a). In a clinical study, Tan-re-qing injection was reported to have therapeutic effects on acute lung injury (ALI), reducing the levels of the serum inflammatory factors TNF-α, IL-6 and IL-8, delaying the progress of systemic inflammatory response syndrome (SIRS), slowing down the progress of SIRS and reducing the degree of respiratory distress (Yang et al., 2010). Postresuscitation lung injury was attenuated by Shen-fu injection *in vivo* by inhibiting lung cell apoptosis and improving energy metabolism and antioxidant capacity (Zhang et al., 2012). In addition, through *in vivo* and clinical trials confirmed that miRNA in honeysuckle decoction can be effectively absorbed through drinking, and effectively inhibits SARS-CoV-2 replication *in vivo*, and accelerates the negative conversion of COVID-19 patients (Zhou L.-K. et al., 2020).

COVID-19 belongs to the category of “epidemic” in TCM because of its strong infectivity and fast spread with the characteristics of “dampness, poison and epidemic”, whose main site is the lung, involving the spleen and stomach (Luo et al., 2020a). Cold-dampness and dampness-heat in the lung symptoms are shown in mild cases of COVID-19, dampness toxin and cold-dampness obstructing the lung symptoms in ordinary, epidemic toxin obstructing the lung and blazing of both qi and nutrient symptoms in severe, and internal block and external collapse symptoms in critical (Ho et al., 2020). In TCM theory, the properties of herbal drugs include four fundamental characters: cool, cold, warm, and heat and five fundamental tastes: pungent, sweet, bitter, sour, and salty. Among the formulas of COVID-19 issued in China, the four flavors of the drugs for COVID-19 are mainly warm, cold, and flat, the five tastes were mainly bitter, hot, and sweet, and the meridians were mainly lung, stomach, and spleen (Zhou Z. et al., 2020). The pungent medicines are mostly used in the early stage, which the bitter drugs are valued in the middle and severe stage, and the sweet medicines are mostly found in the recovery stage (Gu et al., 2020). For example, JHG granule, Huo Xiang Zheng Qi powder, Lianhua Qingwen capsule and Xuan Fei Bai Du granule are widely used in mild and ordinary patients, with *Ephedra sinica* Stapf, *Pogostemon cablin* (Blanco) Benth and *Carthamus tinctorius* L. and other pungent herbal drugs*.* XBJ injection, Shen-fu injection and Tan re qing injection are valued in severe and critical patients. It is worth pointing out that Hua Shi Bai Du recipe and QFPDD have the characteristics of mild in nature and taste, having therapeutic effects on all periods. In fact, most of these herbal drugs have the effects of resolving dampness and detoxifying. Among them, *Glycyrrhiza glabra* L., *Scutellaria baicalensis* Georgi, *Ephedra sinica* Stapf, *Pogostemon cablin* (Blanco) Benth, *Carthamus tinctorius* L., *Prunus amygdalus* Batsch and *Magnolia officinalis* Rehder and E.H.Wilson are used more frequently (Gu et al., 2020; Wang C. et al., 2020; Zhou Z. et al., 2020). *Pogostemon cablin* (Blanco) Benth and *Thespesia populnea* (L.) Sol. ex Corrêa are frequently used to treat medical ailments, such as removing dampness (Yang et al., 2016; Lv et al., 2018). *Lonicera japonica* Thunb. and *Glycyrrhiza glabra* L. are popular with efficacy of clearing heat and detoxifying used in TCM (Nomura et al., 2002; Cao et al., 2012). However, presently studies on the treatment of COVID-19 TCM herbal formulas were mostly *in vivo* and *in vitro* studies, retrospective studies and case-control trials; few rigorous randomized controlled trials (RCTs) were carried out; thus, more RCTs should be carried out.

### Integrated Traditional Chinese and Western Medicine for Coronavirus Disease 2019

Due to the different research methods and theoretical systems, traditional Chinese medicine and western medicine have their unique characteristics and advantages. TCM focuses on an overall approach to the analysis of illness and the patient's condition, and carries out diagnosis and treatment from integrative and holistic points of view. Different from TCM, western medicine focuses on the common law of diseases and treats it through analysis. Integrated traditional Chinese and western medicine (abbreviated as “integrated medicine”) for COVID-19 has been successful in China. Respiratory support and circulatory support are important treatment methods in western medicine, while TCM has shown beneficial effects in improving clinical symptoms, immune regulation and organ protection (Huang Y.-F. et al., 2020). Clinical studies have shown that integrated medicine tends to decrease the mortality rate of SARS (Zhang et al., 2004) and is superior to western medicine in improving the clinical symptoms of COVID-19 patients (Zhang et al., 2020). Nelfinavir in combination with spilanthol to treat COVID-19 was found to enhance its ability to control viral proliferation and improve the risk of disease progression and transmission (Ohashi et al., 2020). A systematic review and meta-analysis of the efficacy and safety of integrated medicine for COVID-19 including 11 studies from six databases revealed that the overall response rate (*p* = 0.000), cure rate (*p* = 0.002), severity illness rate (*p* = 0.012), hospital stay (*p* = 0.002) and clinical symptom improvement rate (*p* < 0.05) were better than those of western medicine alone (Liu M. et al., 2020). Another systematic review and meta-analysis showed that integrated medicine significantly improves the total effective rate, cough symptom disappearance rate, and sputum production symptom disappearance rate (Ang et al., 2020b). Therefore, integrated medicine can improve the clinical efficacy and has good prospects.

### Application of Modern Science and Technology in the Treatment of Coronavirus Disease 2019 With Traditional Chinese Medicine

Due to the limitations of P3/P4 experimental conditions, the use of computer network technology has increased in drug research and development, such as network pharmacology, artificial intelligence, and CRISPR technology, which can accelerate drug research and development, reduce research costs and save research resources.

#### Study on the Mechanism of Network Pharmacology in Traditional Chinese Medicine

Network pharmacology, a new discipline based on the theory of systems biology and biological system network analysis, clarifies the mechanisms of multi-component, multi-target, and multi-pathways. Both TCM and network pharmacology emphasize a comprehensive understanding of diseases. Kaempferol, baicalein and oroxylin A in JHG might regulate various signaling pathways (such as PTGS2, BCL2 and CASP3) by binding to ACE2, thus playing a therapeutic role in the treatment of COVID-19 (Jimilihan, 2020). The mechanism of Lianhua Qingwen in the treatment of COVID-19 may be to improve human immunity by participating in T-cell and B-cell receptor signal transduction and natural killer cell-mediated cytotoxicity, as well as to exert anti-inflammatory effects through Fc epsilon RI, ErbB, ErbB, MAPK and other signal pathways (Ye et al., 2020). The protein interaction map screened 66 potential targets (e.g., Nsp1, Orf10, Spro, and Npro) and 69 potential drugs (e.g., chloroquine and azithromycin) of SARS-CoV-2 (Gordon et al., 2020).

#### Artificial Intelligence Helps Coronavirus Disease 2019 Traditional Chinese Medicine Research

Artificial intelligence (AI) has gradually become an important factor affecting the development of the pharmaceutical industry and has been widely used in numerous medical fields, such as intelligent medical robots, voice intelligence diagnosis and treatment, medical and health management systems, and drug research and development. AI is widely used in the prevention and control, diagnosis and treatment of COVID-19. Regarding prevention and control, through establishing a scientific model of an infectious disease transmission mechanism, a significant effect of community prevention and control policies will occur when 40–60% of the group abide by the policy. Additionally, a travel restriction policy can effectively reduce the risk of disease transmission caused by an insufficient proportion of people who abide by the policy (St-Onge et al., 2020). In terms of diagnosis, an AI model using CT images to assist in the diagnosis of COVID-19 was successfully established, with a total accuracy of 83% and that is rapid and efficient (Wang S. et al., 2020). Concerning treatment, AI is mainly used in the design and screening of small-molecule drugs. Baritinib, an effective immunosuppressant approved by the U.S. Food and Drug Administration (FDA), has been predicted to reduce the ability of novel coronavirus to infect lung cells (Richardson et al., 2020). Glycyrrhizin significantly inhibits the replication of SARS-CoV-2 in Vero E6 cells by imitating type I interferon (EC_50_ = 2.39 μmol/l) (Zhu J. et al., 2020). Professor Li Hua’s research team proved that *Platycodon grandiflorum* saponins D and baicalin had high affinity with PLpro, and andrographolide and its derivatives with 3CLpro and RdRp by gene sequence comparison, homologous modeling and computer virtual target screening (Wu et al., 2020b).

#### Other Modern Science and Technologies

Many other modern science and technologies are also used in COVID-19 in addition to network pharmacology and AI. Molecular docking techniques have been used to predict the ACE2-binding abilities of baicalin, scutellarin, hesperetin, nicotine, and glycyrrhizin, which are possible therapeutic agents for COVID-19 (Chen and Du, 2020a). A prophylactic antiviral CRISPR in human cells (PAC-MAN) for viral inhibition has been developed that can effectively degrade RNA from SARS-CoV-2 sequences and live influenza A virus (IAV) in human lung epithelial cells (Abbott et al., 2020). The proteome and metabolite profiles of 99 serum samples (53 control vs. 46 COVID-19) were obtained by high-resolution mass spectrometry, and the results showed that molecular changes in the serum of patients with severe COVID-19, such as macrophage dysfunction, platelet degranulation, the complement system pathway and global metabolic inhibition (Shen et al., 2020). Moreover, Watanabe et al. (2020) used a site-specific mass spectrometric approach to map the glycan-processing states of SARS-CoV-2 S protein. Proteomics revealed changes in the protein levels and pathways in host cells infected with SARS-CoV-2 and demonstrated that SARS-CoV-2 replication in cells could be prevented by inhibiting compounds of these pathways (Bojkova et al., 2020)**.** Single-cell RNA sequencing (scRNA-seq) used to analyze the peripheral immune response of patients with severe COVID-19, the results showed that peripheral immune cell phenotype was reconfigured and peripheral blood monocytes and lymphocytes did not express a large number of pro-inflammatory cytokines indicating that they were not involved in the cytokine storm (Wilk et al., 2020). Using RNAscope technique, it was found that the expression levels of ACE2 and TMPRSS2 in nasal epithelial cells were significantly higher than the lower respiratory tract indicating the nasal susceptibility to SARS-CoV-2. Furthermore, the increase of CD68^+^ and CD163 + macrophages in inflammatory infiltrating pulmonary parenchyma cells was observed based on RNAscope technique in a rhesus macaque model of SARS-CoV-2 infection (Chandrashekar et al., 2020).

Modern science and technology have not only played important roles in fighting the COVID-19 epidemic but have also provided an objective theoretical basis for the clinical application of TCM.

## Difficulties and Solutions Faced by the Modernization of Traditional Chinese Medicine

TCM is not only one of the greatest treasures of ancient Chinese science but also the key to the treasure house of Chinese civilization. Different from western medicine, a very important point is that TCM upholds the theory of “treat disease before it arises.” The theory emphasizes disease prevention, as well as early diagnosis and treatment to prevent aggravation of the disease. TCM has been administered early, widely and in many participants, reflecting its unique value in the prevention and control of COVID-19 epidemic. TCM can inhibit the growth of the virus in the body, relieve patients’ symptoms and prevent serious development of the disease with the functions of clearing heat, removing dampness and detoxification (Medicine NAoTC, 2020e). According to Chinese news reports, TCM has been used in more than 90% of COVID-19 patients (Liu, 2020), and the effective rate of integrated medicine is more than 92% in Beijing (Medicine NAoTC, 2020a). Because of the lack of convincing and strict experimental data, the therapeutic effect and safety of TCM have been questioned and is the main problem in the development and modernization of TCM.

Presently, most clinical studies of TCM are case reports and case-control studies, with a lack of rigorous and scientific randomized controlled trials (RCTs). Thus, the findings lack strong scientific evidence to be widely recognized. To address this problem, the TCM Clinical Evidence database (http://www.tcmevd.com/evidence/index) has been launched on April 22, 2020. Additionally, attention should be focused on standardized clinical studies of TCM, and communication between evidence-based medicine fields domestically and abroad should be strengthened. Moreover, the mysterious veil of TCM can be lifted, and the modern medical systems can be enriched and improved with the help of modern science and technology. TCM has many ingredients, unclear effective activity and complex mechanisms; thus, it is difficult to effectively guarantee its safety. Fortunately, these drawbacks can be clarified with modern science and technology, such as network pharmacology, molecular docking technology and data mining (Ye et al., 2020). The process and dynamic changes of TCM *in vivo* can be confirmed through pharmacokinetic and metabonomic studies. AI has contributed to the improvement of the service system of modern traditional Chinese medicine (Wang S. et al., 2020). Importantly, the lack of objective evaluation criteria for the diagnosis and treatment of TCM is a serious problem. TCM diagnosis refers to collecting information on clinical symptoms and signs using four diagnostic processes—looking, listening and smelling, asking and cutting—which are very subjective and closely related to personal experience. Thus, the information in the process of diagnosis should be standardized and digitized, and a scientific diagnostic standard and a systematic evaluation system should be established. TCM is gradually being adopted worldwide because of its unique advantages and efforts by the Chinese, and is expected to make positive contributions to the health of all people globally.

## Conclusion and Prospect

With the outbreak of COVID-19 epidemic, great efforts are being made to understand the pathogenesis and clinical characteristics of the disease to identify effective drugs and vaccines. However, no specific antiviral drugs and vaccines are available currently. Symptomatic and supportive therapies are the main treatment approaches, including general therapeutic measures, antiviral therapy and respiratory support. The history of TCM fighting against epidemics has spanned thousands of years. In China, TCM has been widely and deeply involved in the diagnosis and treatment of COVID-19 and has played a positive role in this war. Treatment using integrated traditional Chinese and western medicines has achieved success, and modern science and technology have made significant contributions. COVID-19 treatment results have shown that TCM is effective in relieving symptoms, improving the cure rate, reducing mortality, and promoting the rehabilitation of convalescent people, without world recognition. Presently, TCM diagnosis and treatment still lack objective evaluation criteria, its efficacy lacks strong scientific evidence, and its mechanisms of action are unknown. To realize the modernization of TCM, we should focus on evidence-based medicine, combined with science technology, and establish standards of diagnosis and treatment. We hope that, through the contribution of TCM, combined with modern technological research and the support of our international counterparts, COVID-19 can be effectively controlled and treated.

## Author Contributions

LL conceived and designed the review; QQ, FH, XHL, XLL, and LL wrote the manuscript; LL, YH, HL, and LC reviewed the paper and provided comments, and all of the authors reviewed the manuscript.

## Funding

This project was supported by the PhD Start-up Fund of Guangdong Medical University (B2019016); Administration of Traditional Chinese Medicine of Guangdong Province (20201180); Science and Technology Special Project of Zhanjiang (2019A01009); Natural Science Foundation of Guangdong Province (2016B030309002); “Group-type” Special Supporting Project for Educational Talents in Universities (4SG20138 G); Basic and Applied Basic Research Program of Guangdong Province (2019A1515110201).

## Conflict of Interest

The authors declare that the research was conducted in the absence of any commercial or financial relationships that could be construed as a potential conflict of interest.
